# Correction: Boiten et al. Pathologically Decreased CSF Levels of Synaptic Marker NPTX2 in DLB Are Correlated with Levels of Alpha-Synuclein and VGF. *Cells* 2021, *10*, 38

**DOI:** 10.3390/cells11040652

**Published:** 2022-02-14

**Authors:** Walter A. Boiten, Inger van Steenoven, Mei-Fang Xiao, Paul F. Worley, Barbara Noli, Cristina Cocco, Gian-Luca Ferri, Afina W. Lemstra, Charlotte E. Teunissen

**Affiliations:** 1 Neurochemistry Department, Clinical Chemistry, Amsterdam UMC, 1081 HV Amsterdam, The Netherlands; i.vansteenoven@amsterdamumc.nl (I.v.S.); c.teunissen@amsterdamumc.nl (C.E.T.); 2 Department of Neurology, Alzheimer Center Amsterdam, Amsterdam UMC, 1081 HV Amsterdam, The Netherlands; a.lemstra@amsterdamumc.nl; 3 Department of Neuroscience, Johns Hopkins University School of Medicine, Baltimore, MD 21205, USA; mxiao1@jhmi.edu (M.-F.X.); pworley1@jhmi.edu (P.F.W.); 4 NEF-Laboratory, Department of Biomedical Sciences, University of Cagliari, 09042 Monserrato, Italy; barbaranoli@yahoo.it (B.N.); crcocco@unica.it (C.C.); ferri@unica.it (G.-L.F.)

The authors want to notify that they have made the following changes to the author list of the paper [1]. Barbara Noli, Cristina Cocco, Gian-Luca Ferri have been added as the co-authors. The author list has been amended. Authors are added who supplied the analysis of VGF used in this paper.

Previous authors and affiliations:

Walter A. Boiten ^1,^*, Inger van Steenoven ^1,2^, MeiFang Xiao ^3^, Paul F. Worley ^3^, Afina W. Lemstra ^2^ and Charlotte E. Teunissen ^1,2^

^1^  Neurochemistry Department, Clinical Chemistry, Amsterdam UMC, 1081 HV Amsterdam, The Netherlands; i.vansteenoven@amsterdamUMC.nl (I.v.S.); c.teunissen@amsterdamumc.nl (C.E.T.)
^2^  Department of Neurology, Alzheimer Center Amsterdam, Amsterdam UMC, 1081 HV Amsterdam, The Netherlands; a.lemstra@amsterdamumc.nl
^3^  Department of Neuroscience, Johns Hopkins University School of Medicine, Baltimore, MD 21205, USA; mxiao1@jhmi.edu (M.X.); pworley1@jhmi.edu (P.F.W.)
* Correspondence: w.a.boiten@amsterdamUMC.nl

New authors and affiliations:

Walter A. Boiten ^1,^*, Inger van Steenoven ^1,2^, Mei-Fang Xiao ^3^, Paul F. Worley ^3^, Barbara Noli ^4^, Cristina Cocco ^4^, Gian-Luca Ferri ^4^, Afina W. Lemstra ^2^ and Charlotte E. Teunissen ^1,2^

^1^  Neurochemistry Department, Clinical Chemistry, Amsterdam UMC, 1081 HV Amsterdam, The Netherlands; i.vansteenoven@amsterdamumc.nl (I.v.S.); c.teunissen@amsterdamumc.nl (C.E.T.)
^2^  Department of Neurology, Alzheimer Center Amsterdam, Amsterdam UMC, 1081 HV Amsterdam, The Netherlands; a.lemstra@amsterdamumc.nl
^3^  Department of Neuroscience, Johns Hopkins University School of Medicine, Baltimore, MD 21205, USA; mxiao1@jhmi.edu (M.-F.X.); pworley1@jhmi.edu (P.F.W.)
^4^  NEF-Laboratory, Department of Biomedical Sciences, University of Cagliari, 09042 Monserrato, Italy; barbaranoli@yahoo.it (B.N.); crcocco@unica.it (C.C.); ferri@unica.it (G.-L.F.)
* Correspondence: w.a.boiten@amsterdamumc.nl

The citation part should be updated to the following version:

**Citation**: Boiten, W.A.; van Steenoven, I.; Xiao, M.-F.; Worley, P.F.; Noli, B.; Cocco, C.; Ferri, G.-L.; Lemstra, A.W.; Teunissen, C.E. Pathologically Decreased CSF Levels of Synaptic Marker NPTX2 in DLB Are Correlated with Levels of Alpha-Synuclein and VGF. *Cells* **2021**, *10*, 38. https://doi.org/10.3390/cells10010038.

The “Author Contributions” statement thus should be updated to the following version:

**Author Contributions**: According to CRediT: conceptualization: C.E.T., A.W.L. and I.v.S.; data curation: W.A.B., B.N., C.C. and I.v.S.; formal analysis: W.A.B.; funding acquisition: C.E.T. and A.W.L.; investigation: M.-F.X., P.F.W., B.N., C.C., G.-L.F. and A.W.L.; methodology: P.F.W., C.C., B.N. and I.v.S.; project administration: C.E.T. and A.W.L.; resources: C.E.T., G.-L.F. and P.F.W.; software: not relevant.; supervision: C.E.T. and A.W.L.; validation: W.A.B. and I.v.S.; visualization: W.A.B.; writing—original draft: W.A.B.; writing—review and editing: C.E.T., A.W.L., M.-F.X., G.-L.F. and P.F.W. All authors have read and agreed to the published version of the manuscript.

The authors have confirmed that the updated authorship meets the ICMJE criteria, the guidelines of which are followed by this journal. We apologize for this error and state that the scientific conclusions are unaffected. The original article has been updated.
