# Supplementary material for: Pathologically Decreased CSF Levels of Synaptic Marker NPTX2 in DLB Are Correlated with Levels of Alpha-Synuclein and VGF
Source: Cells. 2020 Dec 29;10(1):38. doi: 10.3390/cells10010038 (PMC7824459; doi:10.3390/cells10010038)
Supplement: Supplementary file 1 [file cells-10-00038-s001.pdf]

Supplementary data

Supplemental Table S1: *Mean and standard deviation used to calculate the z-scores of the cognitive domain tests.*

		Mean	SD
Memory	RAVLT immediate recall	43.4	9.5
	RAVLT delayed recall	8.9	2.8
	Visual association test A	11.7	0.7
Attention	Digit span forward	13.1	2.9
	Trail making test A	36.1	13.6
	Stroop 1	43.7	7.9
	Stroop 2	59.6	12.1
Executive function	Digit span backward	9.8	2.6
	Stroop 3	1.7	0.3
	Trail making test B	82.8	35.5
	Letter fluency	38.3	11.6
	Frontal assessment battery	17.2	1.2
Language	Dutch Boston naming test	11.9	0.6
	Categorical fluency test	23.8	5.7
Visual spatial	VOSP numbers location	9.5	0.9
	VOSP dot counting	9.8	0.5
	VOSP frat let	19.3	0.9

Supplemental Table S2: *The p-values of two normality tests for the different parameters examined. The 0.200* indicates the lower bound of true significance. In red, p-values below 0.05 are indicated. For the three biomarkers examined in this paper the Ln transformed levels were examined as well.*

	Kolmogorov-Smirnova			Shapiro-Wilk		
	SCD	DLB	AD	SCD	DLB	AD
Age	.200*	.200*	0.121	0.188	0.148	0.452
Education	0.009	<0.001	0.062	0.009	0.003	0.022
Global	.200*	0.081	.200*	0.821	0.006	0.681
Memory	.200*	0.132	.200*	0.325	0.084	0.382
Attention	0.104	0.004	.200*	0.375	<0.001	0.248
Executive function	.200*	.200*	.200*	0.424	0.164	0.953
Language	.200*	0.162	0.005	0.491	0.011	0.003
Visual spatial	0.023	0.165	.200*	0.033	0.032	0.983
MMSE	0.023	0.002	.200*	0.002	0.002	0.824
CSF levels						
Aβ $_{1-42}$	.200*	0.094	.200*	0.507	0.100	0.159
T-Tau	.200*	<0.001	0.001	0.902	<0.001	0.002
p-Tau	0.072	0.049	0.074	0.082	<0.001	0.001
NPTX2	0.004	0.008	.200*	<0.001	<0.001	0.002
α-synuclein	0.107	0.092	0.043	0.439	0.287	0.003
VGF	.200*	<0.001	<0.001	0.162	<0.001	<0.001
Ln transformed						
NPTX2	.200*	.200*	.200*	0.184	0.026	0.904
α-synuclein	.200*	0.111	0.089	0.329	0.138	0.061
VGF	.200*	.200*	0.051	0.651	0.581	0.146

Supplemental Figure S3: *Levels of VGF and α-synuclein observed in SCD, DLB, and AD subjects. Boxes are median an interquartile range, whiskers are the 95% percentiles.*

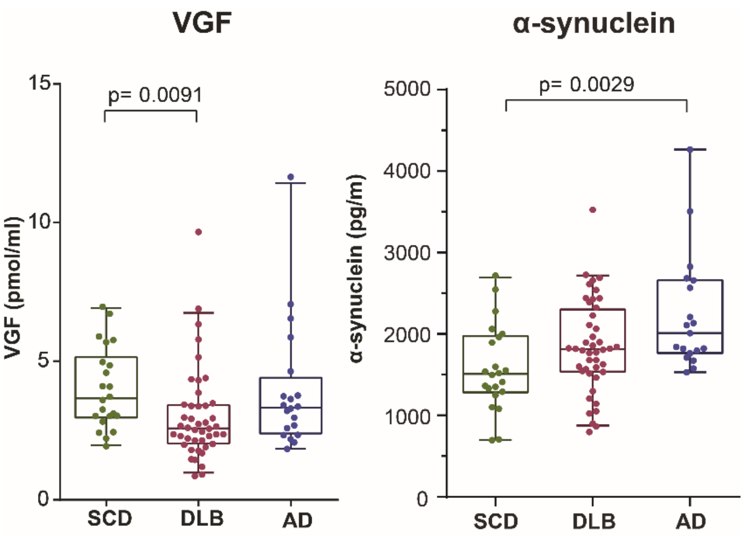

Supplemental Table S4: *Output of linear models comparing the three diagnostic groups, including age as covariate. Models were either tested with SCD or DLB as intercept. The B of the other diagnostic groups are the difference compared to the intercept.*

				Confidence interval	
		B	p-value	Lower	Upper
NPTX2 Ln(pg/ml)	Intercept SCD	6.78	<0.001	5.41	8.15
	Age	0.00	0.856	-0.02	0.02
	AD	-0.49	0.010	-0.87	-0.12
	DLB	-0.58	<0.001	-0.90	-0.27
	Intercept DLB	6.20	<0.001	4.75	7.64
	Age	0.00	0.856	-0.02	0.02
	AD	0.09	0.594	-0.25	0.43
VGF Ln(pmol/ml)	Intercept SCD	0.62	0.229	-0.40	1.65
	Age	0.01	0.169	0.00	0.03
	AD	-0.09	0.546	-0.37	0.20
	DLB	-0.39	0.002	-0.63	-0.14
	Intercept DLB	0.24	0.665	-0.84	1.31
	Age	0.01	0.169	0.00	0.03
	AD	0.30	0.017	0.05	0.55
α-synuclein Ln(pg/ml)	Intercept SCD	6.58	<0.001	5.88	7.29
	Age	0.01	0.040	0.00	0.02
	AD	0.33	0.001	0.13	0.53
	DLB	0.11	0.229	-0.07	0.28
	Intercept DLB	6.69	<0.001	5.94	7.44
	Age	0.01	0.040	0.00	0.02
	AD	0.23	0.012	0.05	0.40

Supplemental Figure S5: *Plots of the Ln transformed values of the three synaptic markers, α-synuclein, NPTX2 and VGF, plotted against one another* ***A, B, and C:*** *Plots of the correlations between the three biomarkers without separating for diagnosis.* ***D, E, F, G, H, and I:*** *Plots of the relations between the three biomarkers separating the different diagnosis.*

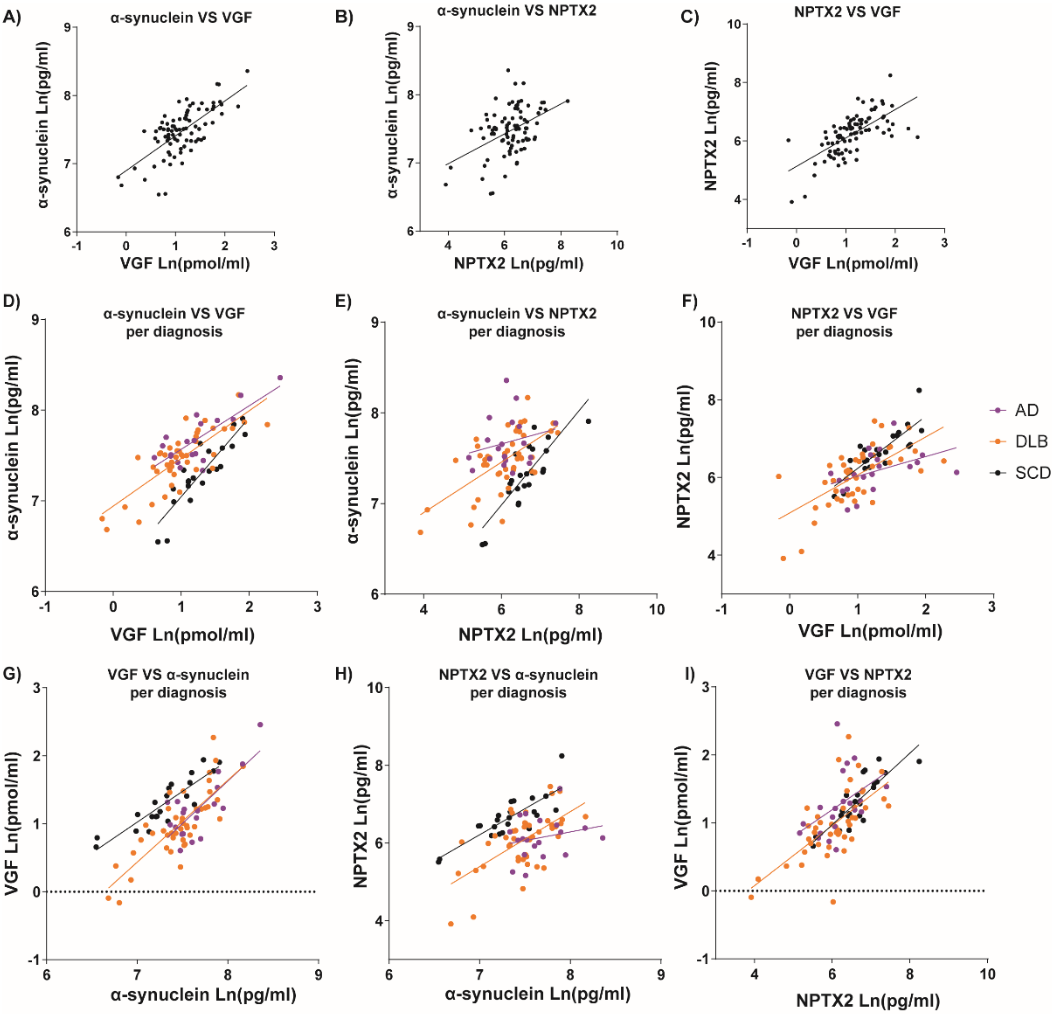

Supplemental Table S6: *Correlations between the biomarkers for both the regular and the Ln transformed data. For the normally distributed data both Pearson and Spearman correlations are given. R.n.n. refers to: redundant non-normal*

		Pearson		Spearman	
	**regular**	r-value	p-value	R-value	p-value
**All**	NPTX2 VGF	r.n.n	r.n.n	***0.709***	***<0.0001***
	α-synclein-NPTX2	r.n.n	r.n.n	***0.337***	***0.0016***
	α-synuclein-VGF	r.n.n	r.n.n	***0.599***	***<0.0001***
	**Ln transformed**				
	NPTX2 VGF	***0.668***	***<0.0001***	***0.709***	***<0.0001***
	α-synclein-NPTX2	***0.437***	***<0.0001***	***0.337***	***0.0016***
	α-synuclein-VGF	***0.696***	***<0.0001***	***0.599***	***<0.0001***
**SCD**	**regular**				
	NPTX2 VGF	r.n.n	r.n.n	***0.819***	***<0.0001***
	α-synclein-NPTX2	r.n.n	r.n.n	***0.804***	***<0.0001***
	α-synuclein-VGF	***0.889***	***<0.0001***	***0.880***	***<0.0001***
	**Ln transformed**				
	NPTX2 VGF	***0.839***	***<0.0001***	***0.819***	***<0.0001***
	α-synclein-NPTX2	***0.827***	***<0.0001***	***0.804***	***<0.0001***
	α-synuclein-VGF	***0.889***	***<0.0001***	***0.880***	***<0.0001***
**DLB**	**regular**				
	NPTX2 VGF	r.n.n	r.n.n	***0.694***	***<0.0001***
	α-synclein-NPTX2	r.n.n	r.n.n	***0.544***	***0.0001***
	α-synuclein-VGF	r.n.n	r.n.n	***0.731***	***<0.0001***
	**Ln transformed**				
	NPTX2 VGF	***0.658***	***<0.0001***	***0.694***	***<0.0001***
	α-synclein-NPTX2	***0.619***	***<0.0001***	***0.544***	***0.0001***
	α-synuclein-VGF	***0.796***	***<0.0001***	***0.731***	***<0.0001***
**AD**	**regular**				
	NPTX2 VGF	r.n.n	r.n.n	***0.608***	***0.0045***
	α-synclein-NPTX2	r.n.n	r.n.n	0.184	0.4503
	α-synuclein-VGF	r.n.n	r.n.n	***0.511***	***0.0255***
	**Ln transformed**				
	NPTX2 VGF	0.445	0.0494	***0.608***	***0.0045***
	α-synclein-NPTX2	0.228	0.3479	0.184	0.4503
	α-synuclein-VGF	0.774	0.0001	0.511	0.0255

Supplemental S7: Quantifying difference between groups using multiple synaptic biomarkers

A: *Depicts a diagram of the different steps taken to examine the relation between the three synaptic markers. 1. Selecting synaptic markers related to similar processes; 2. examine the correlation between these markers and if they were disease specific; 3. Model relation occurring in SCD using multiple linear regression models to predict a selected biomarker. 4. Use the model to calculate the level of the predicted biomarker according to the relation in SCD and determine the difference with the actual value.* ***B:*** *Multiple regression model for predicting α-synuclein form NPTX2 and VGF levels and a model predicting the level of NPTX2 from VGF levels.* ***C:*** *Same figure as Figure 1B, now with a regression line added based on the models form B.* ***D:*** *Difference between SCD based model predicted level of α-synuclein and the true level of α-synuclein. Significance is indicated using a linear regression model shown in Supplemental Table S8.*

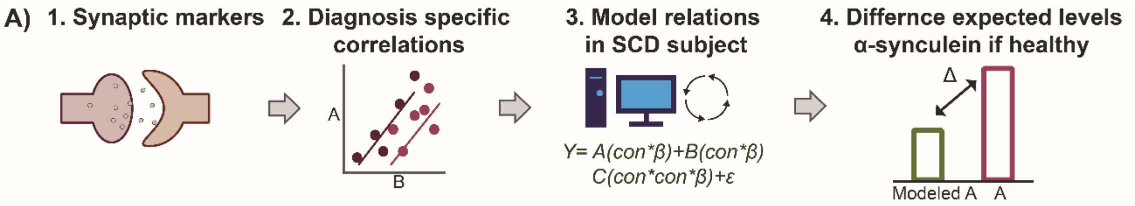

B) Regression models predicting α-synuclein levels

Dependant variable = α-synuclein Ln(pg/ml)

	B	p-value	Lower	Upper	Partial Eta Squared
Intercept	5.280	<0.001	4.103	6.458	0.831
NPTX2 Ln(pg/ml)	0.181	0.119	-0.051	0.414	0.130
VGF Ln(pmol/ml)	0.634	0.002	0.264	1.003	0.419

a. R Squared = .818 (Adjusted R Squared = .798)

Dependant variable =VGF Ln(pg/ml)

	B	p-value	Lower	Upper	Partial Eta Squared
Intercept	-2.184	<0.001	-3.248	-1.121	0.479
NPTX2 Ln(pg/ml)	0.5263	<0.001	0.367	0.685	0.705

a. R Squared = .705 (Adjusted R Squared = .690)

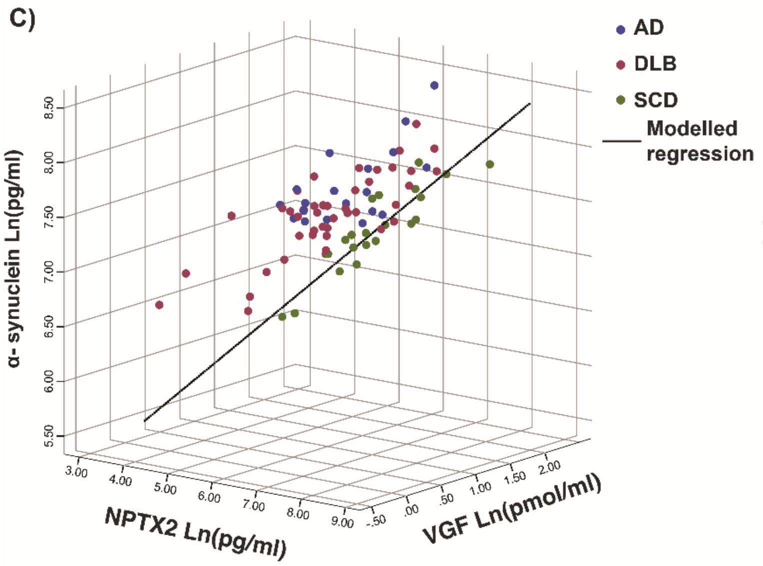

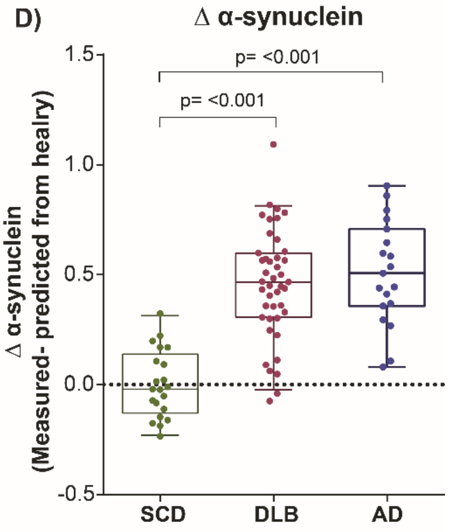

Supplemental Table S8: *Linear model comparing level of Δ α-synuclein. Showing that clear differences in the level of α-synuclein can be observed when corrected for the level of related synaptic markers.*

	B	p-value	Confidence interval Lower	Confidence interval Upper
Intercept SCD	-0.316	0.22	-0.825	0.193
Age	0.005	0.209	-0.003	0.013
AD	0.495	<0.001	0.353	0.637
DLB	0.434	<0.001	0.311	0.558
Intercept DLB	0.118	0.664	-0.421	0.657
AD	0.061	0.329	-0.063	0.185

Supplemental Figure S9: *ROC curves differentiating between SCD and AD, and differentiating between DLB and AD using models of NPTX2, VGF, and α-synuclein CSF levels and age combined, markers combined included all in the model. AUCs and a significance value indicating if it significantly deviates for 0.5 are given.*

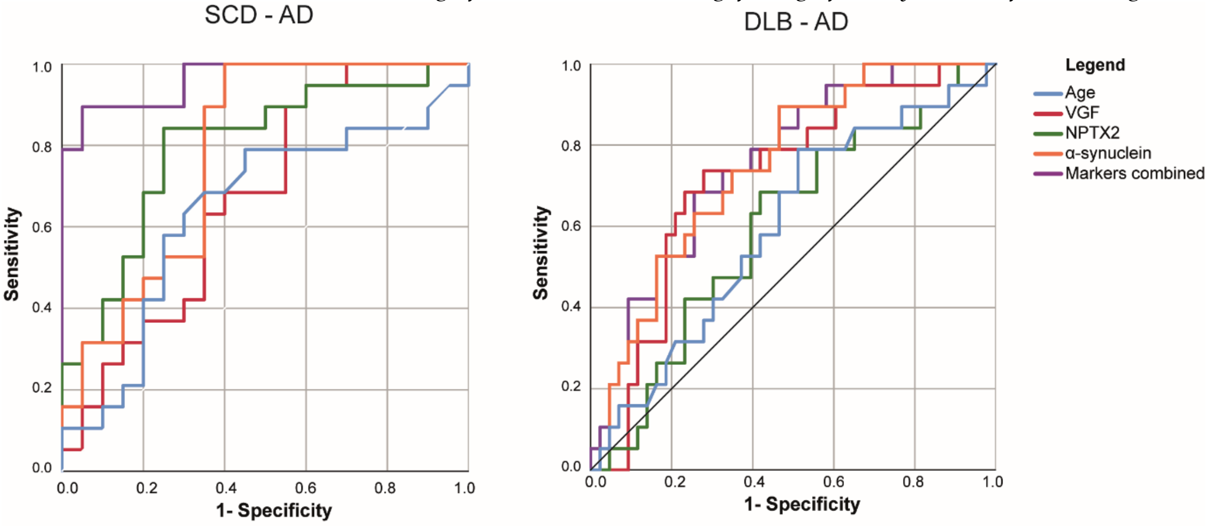

	SCD-AD Area	SCD-AD sig.	DLB-AD Area	DLB-AD sig.
**Age**	0.599	0.216	0.641	0.133
**VGF**	0.671	0.068	0.721	0.006
**NPTX2**	0.795	0.002	0.601	0.208
**synuclein**	0.782	0.003	0.749	0.002
**Combined**	0.963	<0.001	0.759	0.001

Supplemental Table S10: The relation of NPTX2 to cognitive function and cognitive decline in AD patients

For each domain the beta, significance level, and 95% confidence intervals of a model relating NPTX2 to that specific cognitive domain are given. The upper set depict those values for linear models with cognitive function. The lower set shows the values for a linear mixed model for cognitive decline for each domain. Significant values are indicated by bold underline italic fonts

			95% Confidence	
Cognitive function	β	Sig.	Lower	Upper
Global median n=16	0.9391	0.082	-0.138	2.016
Memory n=13	-0.3965	0.722	-2.841	2.048
Attention n=13	1.9776	0.133	-0.730	4.685
Executive =13	0.8532	0.214	-0.591	2.297
Language n=12	-0.1390	0.909	-2.847	2.569
Visual n=10	1.6881	0.377	-2.638	6.014
MMSE n=17	4.0935	0.076	-0.499	8.686
Cognitive decline				
Global median n=9	0.0134	0.363	-0.017	0.044
Memory n=6	0.0487	0.153	-0.020	0.118
Attention n=7	0.0251	0.746	-0.135	0.185
Executive n=8	-0.0181	0.606	-0.090	0.053
Language n=8	0.0010	0.944	-0.028	0.030
Visual n=6	-0.0752	0.247	-0.213	0.062
MMSE n=10	-0.0056	0.956	-0.216	0.205
